# An integrative psychophysiological approach for emotional assessment in outdoor urban settings

**DOI:** 10.3389/fpsyg.2025.1690851

**Published:** 2026-01-27

**Authors:** Chiara Maninetti, Rita Laureanti, Barbara E. A. Piga, Nicola Rainisio, Marco Boffi, Gabriele Stancato, Luca Mainardi, Riccardo Barbieri

**Affiliations:** 1 B3Lab, Department of Electronics, Information and Bioengineering, Politecnico di Milano, Milan, Italy; 2 Urban Simulation Laboratory “Fausto Curti,” Department of Architecture and Urban Studies, Politecnico di Milano, Milan, Italy; 3 Department of Cultural Heritage and Environment, Universitá degli Studi di Milano, Milan, Italy

**Keywords:** electrodermal activity, emotions, heart rate variability, psychophysiology, urban environment

## Abstract

**Introduction:**

Urban environments elicit physical responses from an individual, and also shape their emotional experience. This paper presents an integrative, multidisciplinary methodology combining physiological, psychological and environmental assessments to evaluate citizens' emotional responses to urban spaces.

**Methods:**

The study involved 59 participants walking a predefined route through the Universitá di Milano Bicocca campus while physiological data (ECG and EDA) and psychological self-reports (exp-EIA via mobile app) were simultaneously recorded. Multiple indices of autonomic activity were calculated to map affective states along the route. Polar histograms based on Russell's Circumplex Model visualized dominant emotions at key Points of View (PoVs).

**Results:**

Results revealed significant variations in sympathetic and parasympathetic activation across locations, correlating with environmental features such as green areas, social spaces, or obstructed views. Psychological data aligned with physiological trends, particularly in valence changes.

**Discussion:**

Our findings demonstrate the feasibility of using synchronized psychophysiological measurements to identify emotionally salient urban features, informing evidence-based, people-centered urban design strategies.

## 1 Introduction

The primary goal of urban planning and design is to shape the city experience (Gehl, 2010). The ways in which people move through, feel, and engage with urban spaces affect, among other factors, their emotional state within these environments (Piga et al., 2023). However, the subjective and dynamic nature of these experiences often escapes the standard measures employed in urban planning, which tend to rely on static indicators (e.g., density, land use, and accessibility) and overlook the lived and evolving experience of the inhabitants. Inhabitants actively engage with and interpret urban environments through embodied, emotional, and social processes that shape their experience of a place (Degen and Rose, 2012; Manzo and Devine-Wright, 2021).

A growing body of studies across psychology, neuroscience, architecture, and urban design has demonstrated that understanding how built environments affect people, as reflected in measurable psychological and physiological responses, can contribute to more informed and effective design decisions, particularly in urban regeneration contexts. This aligns with an evidence-based design approach, which advocates for incorporating empirical data on people's experience into the design process to improve outcomes in terms of people and community wellbeing. For example, (Salingaros and Masden 2017) discuss evidence-based architecture as a way to integrate user experience data into architectural choices; (Ulrich et al. 1991) demonstrate how exposure to natural and urban environments influences stress recovery and emotional states; (Berman et al. 2008) show that interacting with natural environments improves cognitive functioning compared to urban environments; (Aspinall et al. 2015) investigated how different urban settings affect psychological states, as measured through mobile EEG, providing actionable insights for urban design; and (Brielmann et al. 2022) review how architectural proportions, biophilic elements, and fractal geometry influence aesthetic experience, walkability, and wellbeing by engaging unconscious neurophysiological processes. As argued by (Niedenthal et al. 2005), embodiment also plays a crucial role in how we process information and respond to stimuli, ultimately influencing quality of life. This perspective supports the integration of embodied experiences into design practice, acknowledging that bodily states shape our interactions with the urban environment. Such work exemplifies how data on lived experience can move beyond analysis to actively shape design in urban planning, architecture, and related domains.

This connects directly to the question on how emotional responses toward urban elements and spatial organization influence people's engagement with the city, framing these experiences as central to urban design considerations. In fact, both the general organization of space and the emotional responses toward urban elements profoundly influence how people interact with the built environment and construct the social, cultural and symbolic significance embedded in places (Russell and Pratt, 1980; Rapoport, 2013). Based on lived experience and environmental cues, people form mental maps and spatial sequences that guide their navigation and understanding of urban space (Lynch, 1996; Arnheim, 2009), emphasizing their active role in interpreting and engaging with the environment through motion. Moreover, quality of life in urban environments involves not only emotional and personal development but also interpersonal connections, fostering a balance between individual expression and community support (Romice et al., 2017).

Positioned within the growing body of research advocating for experiential and participatory paradigms in urban planning and design, this study highlights how experiential qualities of places are not secondary effects but essential drivers of the urban regeneration processes. We argue that cities should be designed explicitly with the experiential dimensions of urban life as one of the central focuses of the design process. In this perspective, building on an evidence-based and interdisciplinary approach, we propose a methodology for assessing the subjective environmental experience centered on physiological and psychological data collection for urban design purposes through direct assessment of *in-situ* emotional responses. The outcomes measure, map, and represent how people actually experience urban spaces, providing insights that can support people-centered design decisions.

### 1.1 Emotional appraisal in the urban context

Emotional responses to stimuli of various nature are typically collected in two ways: through self-assessment questionnaires, and through the measurement of physiological variables such as Electrodermal Activity (EDA), Heart Rate Variability (HRV), and EEG recordings.

A large proportion of existing research on the topic employs psychological and physiological measures to evaluate how specific elements (i.e., greenery or traffic) affect the perception of urban spaces (Resch et al., 2020; Pykett et al., 2020). Previous studies (Fathullah and Willis, 2018; Zhang et al., 2021) suggest, for example, that green natural spaces have a positive impact on stress recovery and mental fatigue, while highly fragmented environments (which are characterized by a high percentage of relatively small elements in the scene) tend to have a depressive effect.

(Fathullah and Willis 2018) explored the option of using emotional data to enable citizens to participate more actively in the urban planning process. Although their study featured a limited pool of experimental subjects, and only qualitative analysis of physiological signals (specifically, electrodermal activity, or EDA), it demonstrated the value of including psychophysiological data in an urban planning process that empowers citizens. The work of (Kim et al. 2020) focused on applying saliency detection analysis on a dataset of EDA, gait patterns, and blood volume pulse (BVP) signals recorded during urban exploration to identify “physical disorders,” elements in urban environments that cause physical or emotional discomfort to pedestrians.

In contrast, (Zhang et al. 2021) used continuous monitoring of electroencephalographic signal (EEG), heart rate (HR), electrodermal activity, and subjective evaluations to evaluate the emotional responses of 29 subjects to the presentation of panoramic fish-eye photographs that depicted urban streets in Beijing. The images were semantically segmented, the elements of the street were classified according to color, and the indicators of the visual pattern were extracted; the psychophysiological responses of the subjects were then analyzed together with the street scenes to the configuration and color of the environment. The authors found that vegetation, and in general greenish colors, as well as diversity and reduced fragmentation of urban scenes positively affected emotions, suggesting a neurophysiological basis for the form-function architectural dichotomy.

In 2022, (Zhang et al. 2022) conducted a study to assess the physiological responses of citizens to potential stressors located at specific points in Copenhagen. Physiological responses were recorded together with GPS tracking and photographs that were segmented to identify elements of the scene. The authors found that traffic and overcrowding are major stressors in urban environments. Furthermore, context plays an important role in determining the emotional reaction to a specific urban element; for example, vegetation, which is typically associated with reduced stress, can act as a stressor instead when it obstructs visibility.

The literature also includes studies addressing the subjective emotional effects that urban environments exert on people, adopting a purely psychological rather than psychophysiological perspective. In these works, natural settings in urban contexts play a central role (Vitale and Bonaiuto, 2024), as they are generally associated with positive emotions such as happiness and joy, although demographic, social, and economic variables may mediate these effects in different contexts (Syamili et al., 2023). While much of the literature emphasizes the role of natural elements, other significant urban features have also been explored, highlighting the importance of public urban spaces for positive emotions. For instance, scenic locations have been associated with higher levels of happiness (Seresinhe et al., 2019); similar effects are observed for open spaces (Birenboim, 2018), and in outdoor urban environments where joy tends to be the predominant emotion more consistently than in indoor environments (Bornioli et al., 2025). A growing body of research focuses on pedestrian experiences in urban contexts, demonstrating that the presence of green and blue elements, wide sidewalks, residential or mixed-use streets, and the presence of landmarks significantly enhances positive emotional responses (Sundling and Jakobsson, 2023). Notably, the positive affect experienced during urban walks is not only an automatic response to abstract elements, as it is deeply influenced by personal connections one has with a specific place, its symbolic value in representing the identity of the area, and its connection with local community (Bornioli et al., 2018).

Considering not only the immediate visible surroundings but also the relationship with the broader context, (Ferreira et al. 2016) show that higher levels of affective attachment to one's neighborhood contribute to its emotional appraisal as a more stimulating and relaxing environment, encouraging prolonged walking. (Iwańczak and Lewicka 2020) offer a further interpretative contribution, showing that emotional reactions such as relaxation and irritation are not merely associated with the presence or absence of individual environmental features. Rather, increased relaxation and reduced irritation tend to occur in urban settings that reflect the spatial patterns described by (Alexander et al. 1977), which correspond to typologies of places commonly appreciated by people. Their study provides empirical psychological support for this urban theoretical framework: the greater the number of identifiable patterns present in a space, the stronger the elicited positive emotional reaction.

Recent advancements in this field highlight a growing interest in the dialogue between psychological research and design disciplines. This interdisciplinary approach, involving fields such as landscape architecture, urban planning, and environmental psychology, aims to go beyond the mere evaluation: the goal is to inform design choices, developing processes and tools that incorporate psychological and emotional dimensions into the environmental appraisal. Similarly to the studies mentioned above, several contributions defining psychology-based design criteria have focused on gardens design, emphasizing their potential to elicit positively-toned emotions even in urban settings (Fumagalli et al., 2020). Within this perspective, the variety of planting and the multisensorial stimulation are recognized as key design elements, while from an experiential standpoint, the ability to foster serenity emerges as a crucial quality (Harries et al., 2023).

In more applied terms, these frameworks have guided the evaluation of urban gardens both in their current condition (Harries et al., 2024) and through post-occupancy evaluations (Boffi et al., 2022). These studies highlight the importance of accounting for the variability in emotional and cognitive responses resulting from specific design choices across the intervention area. These results are consistent with the investigation conducted at the city scale in Camden, which highlights that urban parks are not universally associated with positive emotions. Local contextual factors, such as the behavior of the people present, the level of maintenance of the area, and the alignment of available services with user goals, can contribute to a collective emotional experience that also includes negative dimensions, despite the overall positive evaluation (Meenar et al., 2025). Such emotional and spatial variations were observed also in urban contexts without predominant natural elements. (Piga et al. 2023) produced maps making evident the discrepancies emerging in different spots of a neighborhood that is overall associated with positive emotions. The authors conducted an emotional mapping during an experiential walk through an urban campus; their findings showed that positive emotions were more frequently reported in the recently redeveloped area, which included natural elements and social spaces, while negative emotions were more common in traffic-heavy and neglected zones. The same methodology was employed to assess the effects of Nature-Based Solutions (NBS) in a Milanese district: a comparative exploration in virtual reality (current condition) and augmented reality (design simulation) suggested that green and lime tones of local NBS reduce negative emotional responses (Piga et al., 2021).

The integration of psychological and physiological data to inform urban planning decisions has made significant progress in recent years, largely due to advancements in digital technologies. These developments have supported two major directions. First, they have enabled the exploration of emotion-environment relationships in controlled laboratory settings through the use of virtual reality (VR), allowing for greater experimental control (Zhang et al., 2021). Secondly, they have facilitated *in situ* data collection in real physical spaces, integrating psychological and physiological responses with georeferenced information. A key focus of these studies has been the optimization of measurement tools, aiming to move beyond the prototype stage often characteristic of academic research, and toward more practical applications (Resch et al., 2015).

With this perspective, the creation of an integrated ecosystems of tools becomes crucial for aligning physiological, psychological, and geographic data. This integration allows for the investigation of emotional experiences in urban contexts between specific user groups with varying needs, such as pedestrians and cyclists (Resch et al., 2020), which can be repeated more easily over time. These efforts are embedded in a broader agenda that promotes citizen science and participatory processes in urban transformation. The goal is not limited to collecting data through a “people as sensors' approach; rather, it seeks to develop methodologies that not only inform urban professionals and academic researchers but also support citizens in becoming more informed and aware. This is achieved by using accessible tools and presenting results in formats that are understandable even to non-experts (Pykett et al., 2020; Rainisio et al., 2024). The study presented in this paper aims to test a methodology that responds to these emerging needs.

## 2 Materials and methods

### 2.1 Experimental protocol

The project is part of the Multilayered Urban Sustainability Action (MUSA), an Innovation Ecosystem based in Milan that aims at developing a new sustainability model for the cities of the future. In particular, Spoke 1 of MUSA is focused on urban regeneration, addressing issues such as renewable energy sources, mobility, and urban planning. The Universitá di Milano Bicocca (UNIMIB) campus has recently undergone renovation efforts in its main square, Scienza square, providing an opportunity to conduct a two-step pilot study to test an experimental protocol for evaluating the psycho-physiological reactions of citizens to the urban environment before and after the intervention. The article presents the first step of evaluation before transformation.

The Bicocca district, located in the northern part of Milan, was originally developed as the main industrial hub of Pirelli company, which shaped its spatial and social identity throughout the 20th century. Following the decline of manufacturing activities in the 1980s, the area underwent a major urban regeneration process, resulting in a mixed-use neighborhood that now integrates academic, cultural, residential, and commercial function. The UNIMIB campus plays a central role in this transformation, both spatially and functionally.

The case study focuses on a ten-minute walk from the Greco-Pirelli train station to Scienza square, one of the campus's central public spaces. The square has a distinctly hardscape character and is framed by four symmetrical six-story academic buildings, defined by a regular geometry and a consistent architectural typology. The central sections of the northern and southern buildings feature ground-level apertures that connect the square to the surrounding urban fabric. Despite its large size, the space offers minimal vegetation and limited street furniture; at each corner, green areas are recessed below the plaza level (–1) and remain inaccessible to the public.

The experimental protocol consisted in a guided walk along a predefined path throughout the Bicocca neighborhood, while wearing a sensorized apparatus that recorded the subject's physiological activity through the acquisition of the following signals: Electrodermal Activity, through electrodes placed on the third phalanx of the index and ring finger of the non-dominant hand, and Electrocardiogram (ECG) recorded via three electrodes placed on the left shoulder, right shoulder, and abdomen. The signals were acquired using a Thought Technology Procomp Infiniti 5 system and the BioGraph Infiniti suit running on an Acer Enduro N3 laptop. The sampling frequency was set at 256 Hz for both signals.

**Figure 1:**
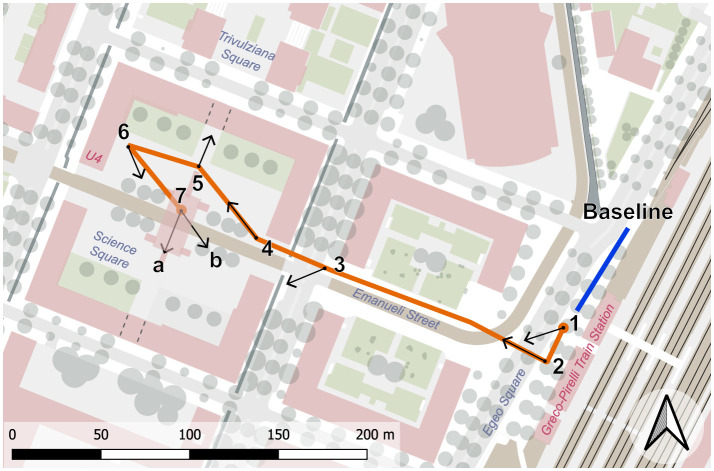
The guided walk consisted of one initial baseline acquisition points, and seven observation points (Points of View). Note that the final point, denoted as “Tramway Stop,” includes two targets that the subjects were asked to treat as two separate observation points.

**Figure 2:**
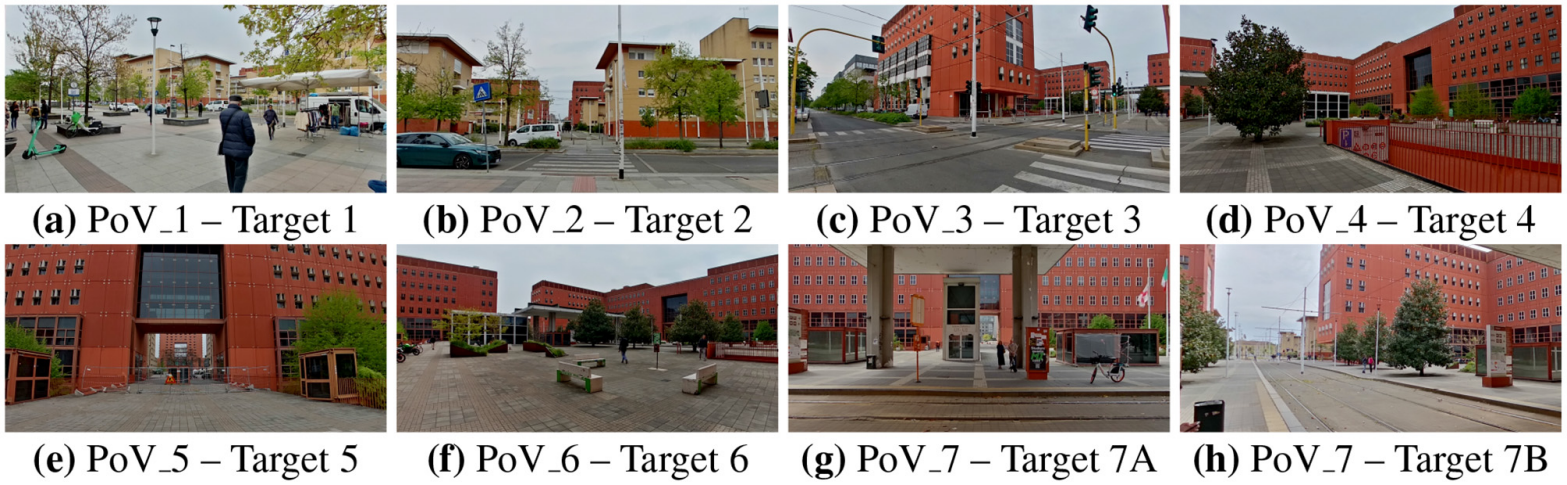
Photographs taken by one experimental subject at each of the selected PoVs. PoV_7 (*Tramway Stop*) is characterized by two targets and has therefore been photographed twice. **(a)** PoV_1–Target 1; **(b)** PoV_2–Target 2; **(c)** PoV_3–Target 3; **(d)** PoV_4–Target 4; **(e)** PoV_5–Target 5; **(f)** PoV_6–Target 6; **(g)** PoV_7–Target 7A; **(h)** PoV_7–Target 7B.

The route and the sight directions were selected to simulate the experience of a person walking from the Milano Greco-Pirelli train station to the classroom and office buildings (see Figure 1 for references). An initial baseline acquisition point and seven Points of View (PoVs) along the path were selected by a team consisting of urban planners and environmental psychologists. Each PoV was characterized by a single direction of view (Target), except for PoV 7 which included two separate Targets labeled as 7A and 7B representing the perspectives from which participants were expected to observe the scene. This choice aimed to investigate whether, despite standing in the same physical location, the psychological experience varied depending on the direction of view. The distinction between PoV and Target represents a specific feature of the exp-EIA methodology, which in previous studies has demonstrated the ability to discriminate between different psychological effects within the same location (Boffi et al., 2022). This results in the analysis of seven PoVs and eight Targets (see Figure 2 and Table 1).

**Table 1:** Points of view and targets.

**Points of view**	**Name**	**Description**	**Targets**
1	Café	Outdoor café outside the Greco-Pirelli train station entrance	1
2	Train station	Piazzale Egeo square in front of the train station	2
3	Crosswalk	Traffic light at the crossing into the university block	3
4	Square entrance	View from entrance of the Scienza Square	4
5	Archway passage	Archway passage view to Piazza Trivulziana	5
6	Square corner outlook	View from the North-West corner of the Scienza Square	6
7	Tramway stop	Covered platform at the tramway stop	7A and 7B

The walk began at the outdoor café located immediately outside the Greco-Pirelli train station entrance (PoV_1). The second point of interest was located in front of the train station, facing Egeo square, that is directly in front of the station (PoV_2). After crossing this square, participants continued along Via Emanueli, walking on the right-hand sidewalk bordering the first built front before reaching the next point of interest at the traffic light crossing into the university block (PoV_3). Upon entering the Scienza square, four additional evaluation points were defined within the square. The first stop (PoV_4) was positioned at the entrance to the square, where the spatial opening first becomes visible upon entry. The second (PoV_5) was located in the northern section of the plaza, beneath the central aperture of the building that faces the neighboring Trivulziana square, creating a framed visual corridor toward the adjacent space; at the time of the experiment, this location was characterized by a metallic fence demarcating the starting point of the construction site for the renovation works, which partially alters the visual connection toward Trivulziana square. The third point in Scienza square (PoV_6) was at the northeast corner of the square, near the entrance to the U4 university building, oriented toward the few benches on that side of the square. The final spot (PoV_7) was under the roof of the tram stop platform and was characterized by two subsequent directions of view: the first looked straight ahead to the other side of the street (Target 7A), while the second faced the tram arrival direction (Target 7B). This spatial sequence of the square, i.e., transition zone, open square, framed passage, corner perspective, and waiting point, was designed to explore the physio- psychological modulations elicited by moving through urban spaces with different spatial and experiential properties.

Participants were instructed in advance to stop walking at each designated PoV along the route and immerse themselves in the atmosphere of the place, focusing on the sounds, visuals, smells, and sensations the environment evoked. After this familiarization period, they were instructed to use the City Sense mobile application to take a picture of the location along the PoV's specified Target and to complete a brief psychological survey. The app integrates the exp-EIA methodology, enabling the combination of spatial behavior, specifically users' geographic position (PoV) and viewing direction (Target) as recorded by the mobile device, with their responses to the questionnaire items (Piga et al., 2021, 2023). The method provides psychological and behavioral information in a unique dataset; it also includes the timestamp, which allows us to integrate the corresponding physiological data. The initial baseline acquisition point was necessary to acquire baseline physiological measures of subjects in a resting state. Here, study participants were shown an emotionally neutral video depicting a scene of marine wildlife. The subjects observed the first minute of the video sitting in an outdoor shaded area at the beginning of the route, while the second half of the baseline was recorded while the subjects were standing in an erect position. Since the protocol included both stationary segments (during which participants stood still at each PoV) and Walking Sections (WS), the signals acquired during the walk from the baseline acquisition site to the first PoV were used as a baseline for normalization of the walking segments of the protocol.

Fifty-nine subjects (mean age 26 ± 5.1 years, 32 men) with various degrees of familiarity with the neighborhood were selected among the general student population to minimize the potential influence of age range and academic background. The sample was balanced with respect to gender and familiarity with the neighborhood. After signing the written consent form, the subjects were fitted with the experimental recording apparatus and given instructions about the route they were to follow and the tasks to complete. Subjects completed the route one person at a time, followed at a distance by trained operators who recorded environmental measures such as weather data (whose collection and analysis are outside the scope of this article and have therefore been omitted) and ensured that the person walked along the correct path. The operators did not interact with the subjects in any other way. The study was carried out according to the Declaration of Helsinki guidelines and approved by the Institutional Ethics Committee of Politecnico di Milano (protocol code 7/2019 on 18 March 2019, and 15/2020 on 15 July 2020).

### 2.2 Physiological assessment of emotion

The final two Points of View (Tram South and Tram East) were were merged into a single PoV for the physiological data analysis. This decision was made due to practical difficulties in reliably separating the corresponding physiological recording segments for the two viewpoints.

#### 2.2.1 Signal processing

Data analysis was performed in Matlab R2023b and Python 3.10.14.

The ECG signal was filtered with a third-order Butterworth filter with a passing band of 0.5 to 50 Hz, to remove power line interference, baseline drift, and other superimposed noise. Beats were automatically extracted using a modified Pan-Tompkins algorithm; the results were manually inspected and erroneous annotations were corrected. The segments of the signal recorded while the subject was walking were particularly difficult to automatically annotate; in several subjects, the heartbeats in these sections required manual selection. The RR signal was extracted as the distance in seconds between subsequent beats. Ectopic beats and artifacts (RR intervals shorter than 200 ms and longer than 3 s) were removed and replaced by shape-preserving cubic piecewise interpolation. Heart rate (HR) was calculated as the reciprocal of the RR series after resampling the signal at a constant sampling frequency of 8 Hz.

The EDA signal was downsampled at a constant sampling frequency of 8Hz. Movement artifacts resulting from the subjects walking and operating their mobile phones were removed using the wavelet method described by (Chen et al. 2015).

#### 2.2.2 Autonomic dynamics assessment

Spectral indices were computed as continuous-time signals sampled at 20Hz after Point Process modeling of the RR series (Barbieri et al., 2005); specifically, the total power content and the power content in the low-frequency (LF) and high-frequency (HF) bands were obtained, as well as the continuous-time sympathovagal balance (LF/HF). A time domain average of each signal was calculated for each subject and for each segment of the recording to obtain punctual indices. Poincaré plots were constructed on the non-resampled RR series, and the following metrics were then obtained: area of the Poincaré ellipsis (ARR), standard deviation along the major axis (SD1) and along the minor axis (SD2), and axis ratio (SD1SD2). The standard deviation of the RR time series (SDRR) and the standard deviation of successive differences (SDSD) were also computed.

The tonic (SCL) and phasic (SCR) components of the EDA signal were then extracted using the cvxEDA algorithm (Convex Optimization Approach to Electrodermal Activity Processing). The parameters τ_0_ and α were selected subject-by-subject as the values that minimized the objective function of the algorithm. The PDA metric was computed as the average of the Phasic Driver of Electrodermal Activity over the interval of interest, after normalization with respect to baseline conditions. $\bar{\text{SCL}}$ was obtained as the mean of the Tonic Skin Conductance during each PoV and WS.

#### 2.2.3 The emotional index

The Emotional Index was introduced by (Vecchiato et al. 2014) to combine the valence and arousal information carried, respectively, by the HR signal and the tonic component of the EDA (Skin Conductance Level, SCL), into one single index. The Emotional Index (EI) is computed according to the arc tangent of the angle β_*EI*_ described by each sampling point inside the arousal-valence Cartesian plane. This value is, in Vecchiato's original formulation, geometrically transformed so as to vary between [−1 1]. We opted to introduce a new index to evaluate the psychophysiological reaction starting from Vecchiato's definition of the β_*EI*_ angle, as it is more easily interpretable and can be referred to the Russel Circumplex model.

The β_*EI*_ angle is computed as follows:


$$\beta_{EI}=\{\begin{array}{ll} \frac{5}{2}\pi -\arctan\left(\frac{{\text{HR}}_{z}}{{\text{SCL}}_{z}}\right) & \text{if }{\text{SCL}}_{z}\geq 0,{\text{HR}}_{z}\leq 0\\ \frac{\pi }{2}-\arctan\left(\frac{{\text{HR}}_{z}}{{\text{SCL}}_{z}}\right) & \text{otherwise} \end{array}$$ (1)

where HR_*z*_ and SCL_*z*_ denote the *z*-score of the HR and SCL signals, respectively. In this polar version of the Russell's plane, the β_*EI*_ angle of a point with respect to the positive x-axis indicates the specific emotional quality or type of affect being experienced, while the distance *d* from the center signifies the intensity or strength of the emotional experience. Our physiological measurements allowed us to compute the angle β_*EI*_, but not the radial distance *d*, giving us the ability to evaluate the emotional response that was experienced, but not to quantify its intensity.

As a time-varying signal, the β_*EI*_ angle carries information about the temporal evolution of a subject's psychophysiological state. A more synthetic representation is necessary to represent overall emotional reactions during an interval of time (such as the time a subject spent observing a PoV), without losing information about the distribution of the values. Therefore, we identified the psychophysiological Dominant Emotion (DE) experienced during each segment of the walk by building a polar histogram of the calculated β_*EI*_ angles. To that end, we divided the Russell's plane into 16 circular sectors, each corresponding to a core emotion that could be related to the emotion actually felt by participants: “happy,” “elated,” “excited,” “alert,” “tense,” “nervous,” “stressed,” “upset,” “sad,” “depressed,” “lethargic,” “fatigued,” “calm,” “relaxed,” “serene,” and “contented,” respectively, starting from the positive x-axis and moving counterclockwise. The height of each bar of the polar histogram indicates the frequency with which a β_*EI*_ angle fell within the specific sector of the Russell plane. Each experimental segment (i.e., each PoV) had a slightly different duration. Therefore, to make the emotional distributions comparable across segments, we calculated the relative frequency as the percentage of time during which the β_*EI*_ angle fell within the bin corresponding to a specific emotion, divided by the total duration of that segment. Dominant Emotions, or the emotions that are experienced more often during the time interval, correspond to the peak (or peaks) of the polar histogram.

The polar histogram plot of ${\hat{\beta }}_{EI}$ values also allows for a rapid comparison with the results of the psychological measures collected during the experimental protocol. Such results include mean arousal and valence values computed for each PoV that identify a specific point in the Cartesian Russell plane.

The β_*EI*_ signal was computed for each subject after normalization of both HR and SCL time series with respect to their mean and standard deviation measured during the baseline sections of the protocol. For each subject and each section of the protocol, the signal β_*EI*_ was represented in histogram form with bin width equal to 0.01. The Peak Emotional Angle ${\hat{\beta }}_{EI}$ was then extracted as the mid-range value of the bin corresponding to the peak of the histogram.

**Figure 3:**
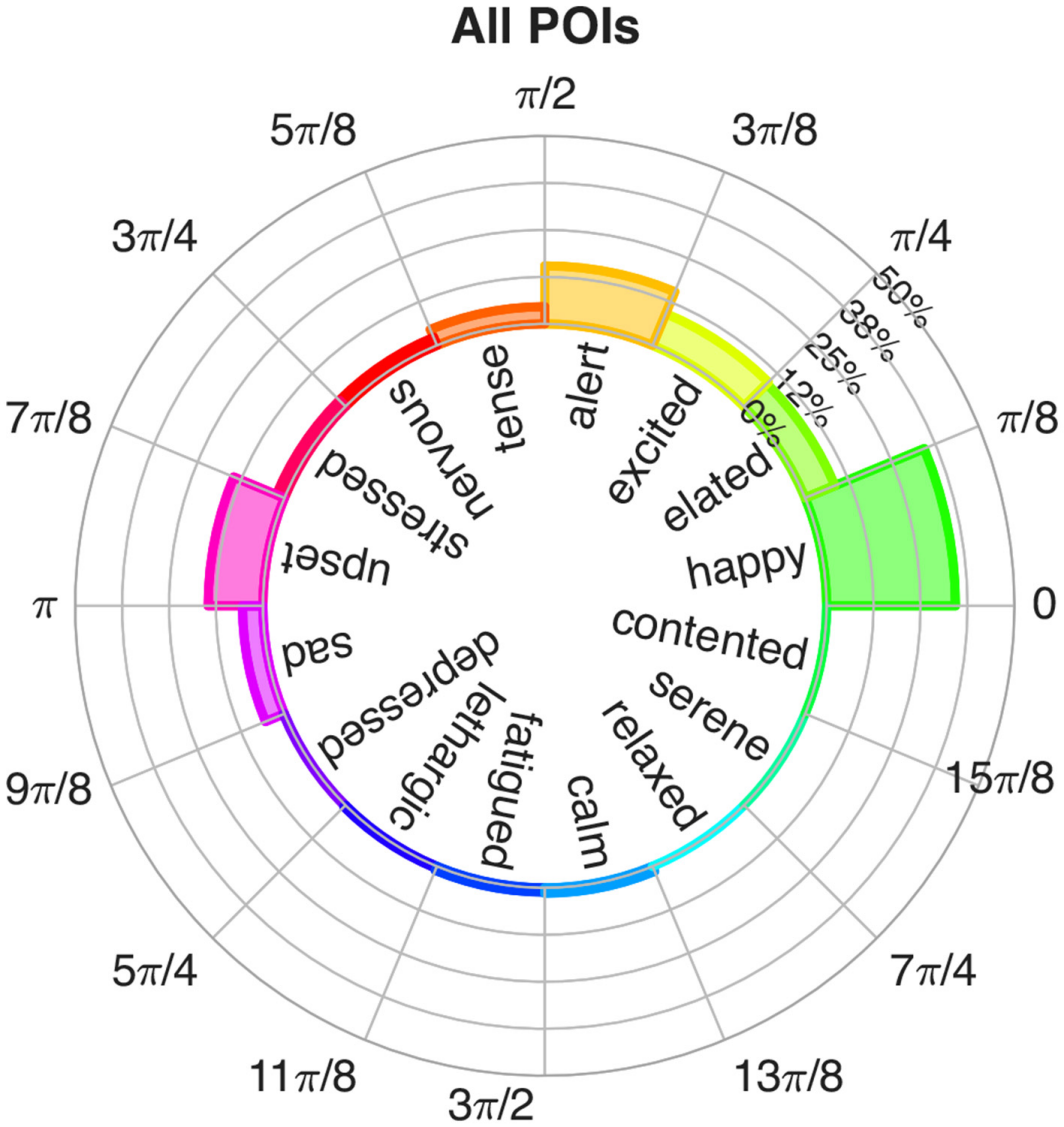
The polar histogram plot of ${\hat{\beta }}_{EI}$ values. Height of the bars represent the relative frequency with which each emotion was recorded during the specific time interval.

The Dominant Emotion of each PoV and WS was computed as follows. First, segments of the β_*EI*_ signal corresponding to the same PoV or WS recorded on each subject were concatenated. The aggregated data series were then used to construct a 16-bins histogram (see Figure 3), with each bin corresponded to one of the 16 circular sectors making up the Russell's circle, and thus one of the 16 discrete emotional states (see Materials and Methods, Section 2.2.3). The histogram counts were then normalized with respect to the total number of samples to obtain the frequency with which each of the 16 standardized emotions was recorded. For each PoV and WS, the Dominant Emotion (DE) was identified as the emotion bin characterized by a histogram count greater than or equal to the 92.5 percentile of all relative histogram counts.

### 2.3 Psychological assessment of emotion

Emotional data were collected using a native digital scale, representing an evolution of the Self-Assessment Manikin (SAM) (Bradley and Lang, 1994). SAM measures two fundamental dimensions of affective experience: valence and arousal, following the circumplex model of affect proposed by Russell to describe perceived environmental qualities (Russell and Pratt, 1980). Following the visual design of the “affective slider” (Betella and Verschure, 2016), a digital version of SAM, the scale of the current study is fully pictorial and optimized for use on digital devices. It includes two separate sliders, each representing a continuum: icons placed at the extremes of each bar symbolize the minimum and maximum levels of valence (displeasure-pleasure continuum) and arousal (deactivation-activation continuum), respectively. Participants indicated their emotional state as a reaction to the environment by moving the cursor along each slider, and the selected positions are automatically converted into numerical values recorded by the application. Following the approach of Russell and Pratt, we consider such values as a support “to provide a conceptual structure that defines the meaning of terms within the domain of the English language that persons commonly use to describe the emotional quality of environments” (Russell and Pratt, 1980, p. 312). According to this perspective, the values of valence and arousal define a region of the Cartesian affective space described by Russell's circumplex model, where each region corresponds to the “core affect” experienced by people. Despite being a partial representation of the whole “prototypical emotional episode,” which also includes antecedents, cognitive processing, behavior and neurophysiological processes, core affect is a fundamental constituent and we rely on (Russell and Barrett 1999) taxonomy to describe it. Therefore, both psychological and physiological “emotions” in the manuscript refer to core affect indirect constructs, derived from continuous measurements rather than participants' explicit naming of emotions, as suggested by the authors (Russell and Barrett 1999).

### 2.4 Statistical analysis

Table 2 provides a list of the physiological indexes that were extracted for each subject and for each segment of the experimental protocol.

**Table 2:** Computed indexes.

**Physiological variable**		**Metrics**	**Description**
RR series	Spectral Indexes	LF/HF	**Sympathovagal balance**. Ratio of sympathetic to vagal modulation.
		LF	**Low frequency power** Reflects mixed sympathetic/respiratory activity.
		HF	**High frequency power** Marker of vagal activity.
		TSP	**Total spectral power**.
	Poincaré indexes	SD1	**Standard deviation in minor axis**. Short-term HRV, related to vagal modulation.
		SD2	**Standard deviation in major axis**. Long-term autonomic fluctuations in HRV.
		SD1SD2	**Axes ratio**. Ratio between short- and long-term HRV.
		SDRR	**Standard deviation of time series**. Global HRV.
		ARR	**Area of the ellipse**. Overall HRV magnitude.
		SDSD	**Standard deviation of successive differences**. Standard deviation of HRV.
EDA signal		PDA	**Phasic driver of electrodermal activity**. Mean amplitude of the estimated sudomotor nerve spikes, related to immediate responses to stimuli.
		$\bar{\text{SCL}}$	**Mean tonic skin conductance level**. Slow-varying component of the electrodermal activity.
Emotional index		$\hat{EI}$	**Peak emotional index**.
		DE	**Dominant emotion**.
		${\hat{\beta }}_{EI}$	**Peak** **β_*EI*_ angle**.

For each segment of the protocol, the distribution of the computed indexes in all experimental subjects was tested for normality using a one-sample KS test. The DE index was excluded from this statistical analysis because it was calculated individually PoV based on the aggregated data collected from all subjects, rather than on a subject-by-subject and PoV-by-PoV basis. Additionally, since the DE index represents an emotional state, it is treated as a categorical variable. Non-Gaussianity was evidenced in all indexes, with the exclusion of SD1SD2: thus, statistical significance was evaluated via Friedman Multiple Comparison tests, with Dunn's correction for multiple comparison. Indexes were compared between PoVs and WSs to identify differences in the psychophysiological responses of subjects across the various PoVs and WSs.

Participants' data collected through the app were spatially clustered, based on their GPS-tracked positions and on the Target framed with their device, using the DBSCAN algorithm implemented via Scikit-learn 0.22 in Python 3.8. This procedure enabled the identification of specific groups of participants according to their spatial exploration patterns. For each spatial cluster, including participants in the same position and looking in the same direction, the mean values of the emotional responses were calculated. The emotional data were then analyzed through three complementary procedures. First, descriptive statistics were used to position each reported emotional state within a Cartesian plane defined by the valence and arousal dimensions; different affective states were visualized using a color-coded scheme corresponding to their coordinates on this plane. Then, emotional responses were integrated with geographic data regarding the participants' locations and visual targets, resulting in a set of isovist maps. In line with the exp-EIA methodology, each partial isovist, defined as the visible portion of space from a given viewpoint directed at a single Target, was colored based on the corresponding emotional experience. Finally, a within-subjects ANOVA was conducted to determine whether emotional responses—measured along the dimensions of arousal and valence—varied significantly depending on the different points of view (PoV) experienced along the path of the participants. The Mauchly's test of sphericity detected violations in the assumption of sphericity for both the arousal (*W* = 0.181, *p* < 0.001) and the valence (*W* = 0.273, *p* < 0.001) dimensions; Huynh-Feldt correction was therefore applied.

Combined analysis of physiological and psychological metrics was performed by computing change scores for each of the considered variables, and calculating the Spearman correlation coefficient between pairs of physiological and psychological change scores.

## 3 Results

### 3.1 Physiological assessment

#### 3.1.1 Autonomic dynamics assessment

**Figure 4:**
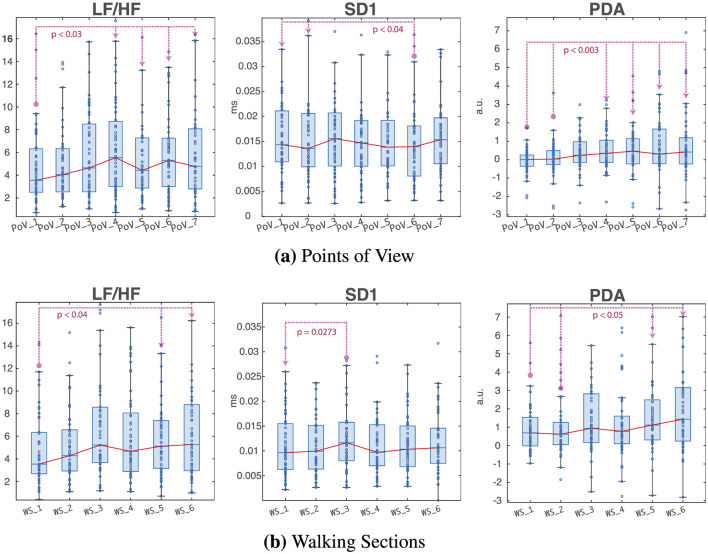
Distributions of some of the metrics that showed statistically significant differences (LF/HF, SD1, and PDA) between **(a)** Points of View and **(b)** Walking Sections. The red line denotes the median trendline, while the blue dots represent individual observations. Dotted lines indicate statistically significant differences between the PoV or WS at the circled endpoint and the ones pointed to by the arrow.

The following metrics of autonomic activity displayed statistically significant difference both between PoVs and between WSs: HF, LF/HF, SD1, SDSD, and PDA. Their distribution throughout the different sections of the protocol is visible in Figure 4.

The high frequency power was found to be significantly lower (*p* < 0.05) in PoV_6 (“Square Corner Outlook”) compared to the other points of view, with the exception of the nearby PoV_4 (“Square Entrance”) and PoV_5 (“Archway Passage”), suggesting a decrease in the subjects' vagal activity in correspondence of this location on the route. The median HF power trendline shows a decrease in PoV_5 (“Archway Passage,” the point before “Square Corner Outlook”), suggesting an even lower median vagal activation than in the “Square Corner Outlook” PoV; however, no statistically significant differences were found when comparing HF power at this location to the rest of the PoVs. Regarding the walking segments of the protocol, the median HF power values remained generally lower than during the observation points, as expected during walking, when the vagal tone decreased consequently to the increased physical demands of the body. In particular, WS_1 (“Cafe → Train Station”) and 3 (“Crosswalk → Square Entrance”) exhibited significantly higher (*p* < 0.02) HF power than other WSs, suggesting that the decrease in vagal activity induced by walking was less prominent during these two segments of walking. In the case of both PoVs and WSs, median trendlines suggest a higher vagal activation during the initial segments of the walk, located closer to the Milan Greco Pirelli train station; parasympathetic activity appeared to decrease once subjects reached the classroom buildings. Notice that the data are not normally distributed and heavily skewed toward the left tail of the distribution; median trends thus do not accurately reflect the mean ranks that were used to evaluate statistical significance. Similar results were found when analyzing the trends in other cardiovascular metrics of vagal activity, namely HF Power (*p*_PoVs_ < 0.05, *p*_WSs_ < 0.02), SD1 (*p*_PoVs_ < 0.04, *p*_WSs_ < 0.03), and SDSD (*p*_PoVs_ < 0.02, *p*_WSs_ < 0.04).

The amplitude of the phasic driver was significantly lower (*p* < 3e − 03) during the first two PoVs (“Café” and “Train Station”) than during the rest of the protocol. Similar results can be observed during WSs, with the first two WSs (“Café → Train Station” and “Square Corner Outlook → Crosswalk”) exhibiting significantly lower (*p* < 0.05) than later segments WS_5 (“Archway Passage → Square Corner Outlook”) and WS_6 (“Square Corner Outlook → Tramway Stop”). These results suggest that the subject experienced higher levels of autonomic activation when they were within the main campus area compared to locations closer to the train station.

Variations in Sympathovagal Balance across PoVs and WSs suggested a similar pattern of initially low sympathetic activation at the beginning of the protocol, which increased as the subject reached the classroom buildings. In particular, statistically significant (*p* < 0.03) variations in Sympathovagal balance were found when comparing PoV_1 (“Cafe”) to PoVs 4 to 7 (“Square Entrance,” “Archway Passage,” “Square Corner Outlook,” and “Tramway Stop”), with PoV_1 exhibiting lower LF/HF than later PoVs. Similarly, the first WS (“Cafe → Train Station”) displayed significantly lower (*p* < 0.04) values than WS_5 (“Archway Passage → Square Corner Outlook”) and WS_5 (“Square Corner Outlook → Tramway Stop”).

#### 3.1.2 The emotional assessment

**Figure 5:**
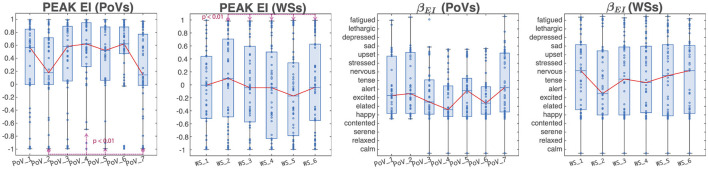
Peak emotional index and peak β_*EI*_ angle, for points of interest and walking sections. The red line represents the median $\hat{EI}$ and ${\hat{\beta }}_{EI}$ values of each PoV and WS.

The Peak Emotional Index displayed statistically significant differences (*p* < 0.01) when comparing PoVs 2 and 7 (“Train Station' and “Tramway Stop”) with PoVs 4 and 5 (“Square Entrance” and “Archway Passage”). Regarding WSs, statistical significance (*p* < 0.01) was found when comparing WS_2 (“Square → Crosswalk”) to WSs 3 to 6 (“Crosswalk → Square Entrance,” “Square Entrance → Archway Passage,” “Archway Passage → Square Corner Outlook,” and “Square Corner Outlook → Tramway Stop”). No statistically significant differences were found when comparing ${\hat{\beta }}_{EI}$ across PoVs and WSs. As evidenced by Figure 5, however, the boxplots displaying the ${\hat{\beta }}_{EI}$ angle distribution suggest that the initial two PoVs (“Cafe” and “Train Station”) elicited a state of excitement in the experimental subjects. PoV_3 (“Crosswalk”), as well as PoV_6 (“Square Corner Outlook”) were characterized by a state of elation/excitement, while PoV_4 (“Square Entrance”) seemed to induce happiness. Finally, PoV_5 (“Archway Passage”) and PoV_7 (“Tramway Stop”) made subjects feel more “alert'. When considering WSs, the first and the last WSs (“Cafe → Train Station' and “Square Corner Outlook → Tramway Stop”) showed a median emotional response indicating nervousness. WSs 3, 4, and 5 (“Crosswalk → Square Entrance,” “Square Entrance → Archway Passage” and “Archway Passage → Square Corner Outlook”) elicited a tense state, while WS_2 (“Square Corner Outlook → Crosswalk”) resulted in a median response closer to excitement.

##### 3.1.2.1 The polar plot representation of emotional states

**Figure 6:**
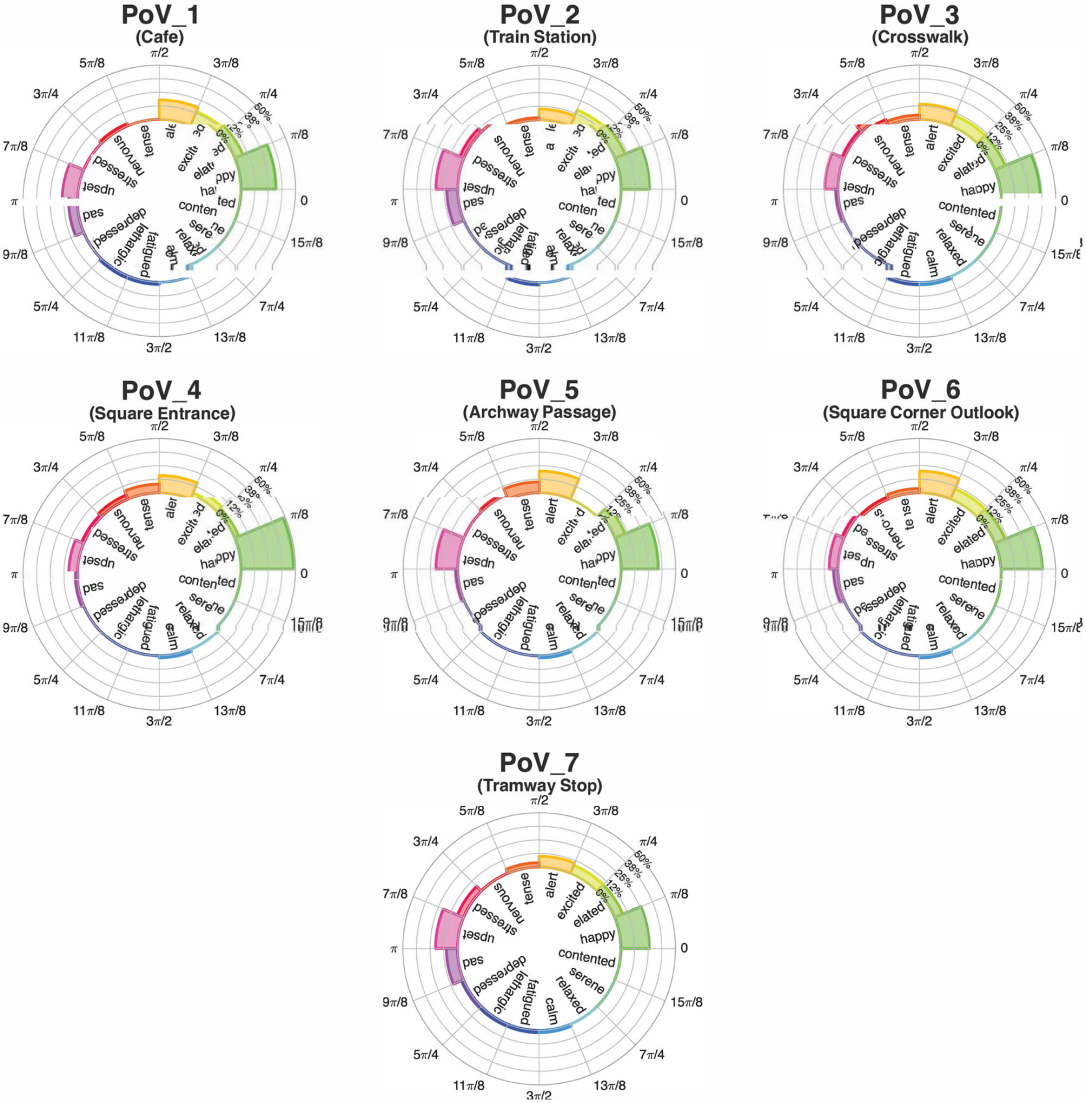
Polar histogram visualization of the Emotional Index, PoV-by-PoV. The height of the bars represent the time-normalized frequency of the measured emotion.

**Figure 7:**
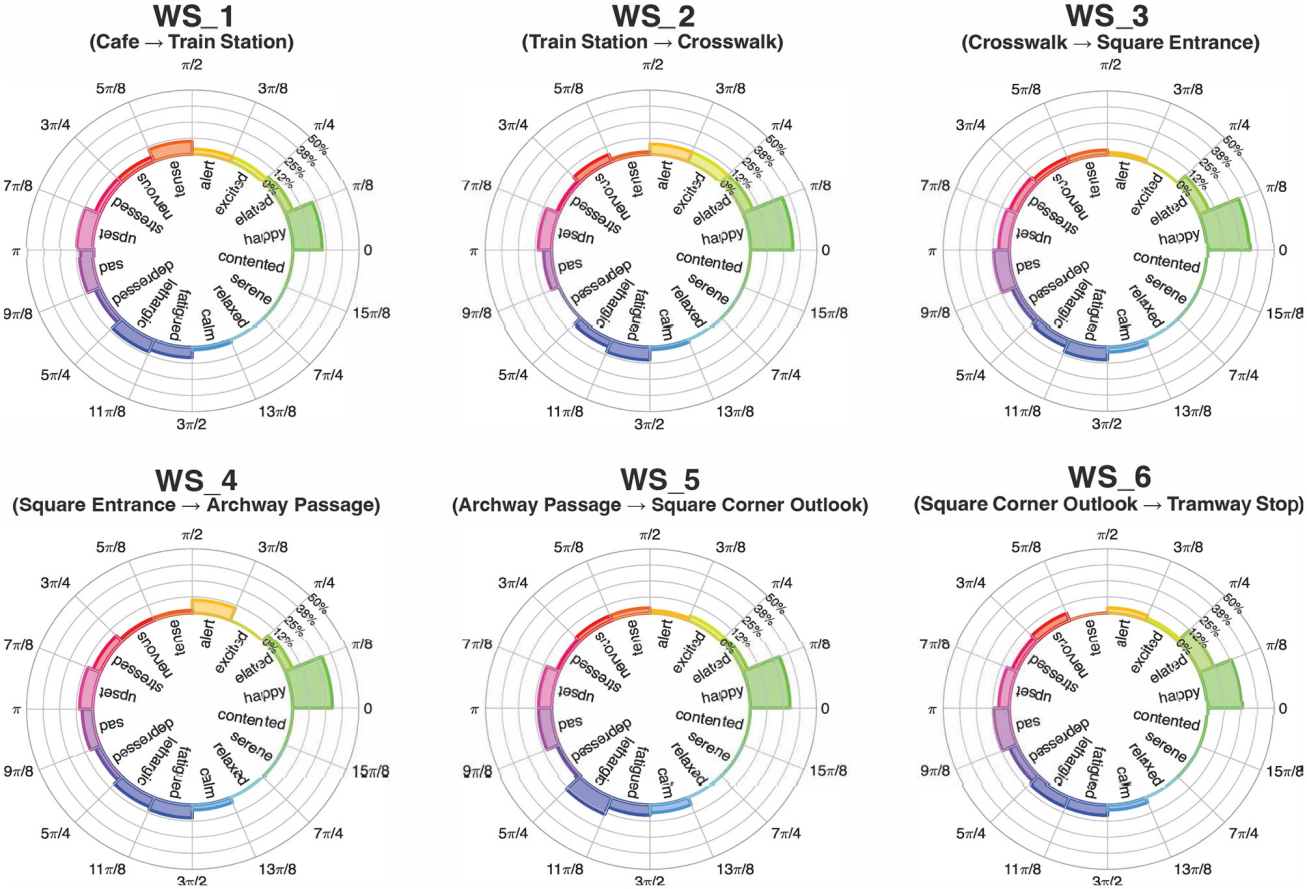
Polar histogram visualization of the Emotional Index, WS-by-WS. The height of the bars represent the time-normalized frequency of the measured emotion.

Figures 6, 7 show the representations of emotions on the polar histograms estimated in each segment of the protocol using the emotional index approach. All PoVs have a strong component in the sector of the Russell plane corresponding to the emotion “happy”; in addition, PoVs “Train Station,” “Archway Passage,” and “Tramway Stop” show a secondary peak in the “upset” sector. WSs showed less dominant peaks: the initial walk toward the first PoV (“Baseline → Café,” which was used as a baseline WS and omitted from statistical analysis, but was included in the representation for visualization purposes) showed a slight dominance of the “excited” and “elated” emotions; gradually, as the walk progressed, the emotions perceived during WSs shifted to the lower arousal “happy” and “contented'. The WSs “Café → Train Station” and “Crosswalk → Square Entrance” displayed further peaks in the “sad” and “upset” sectors. In all 7 PoVs, the average psychological reaction, recorded through the exp-EIA questionnaire sat at the border between the “excited” and “elated” quadrants.

### 3.2 Psychological assessment

#### 3.2.1 Clustered emotional assessment: descriptive statistics and maps

**Figure 8:**
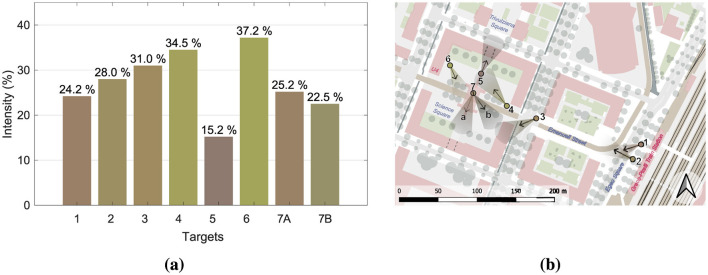
**(a)** Emotional intensities for each PoV. **(b)** Views along the path. Each PoV shows the clustered view direction and position of all participants, corresponding to the recorded average spatial behavior. The colors used for the bar charts **(a)** and cartographic PoVs **(b)** are consistent with the position of the corresponding points on the circumplex model of affect (see Figure 9), where colors vary according to the values of pleasure and arousal. Since the points are all located in the same region of the model, however, the colors appear quite similar.

**Figure 9:**
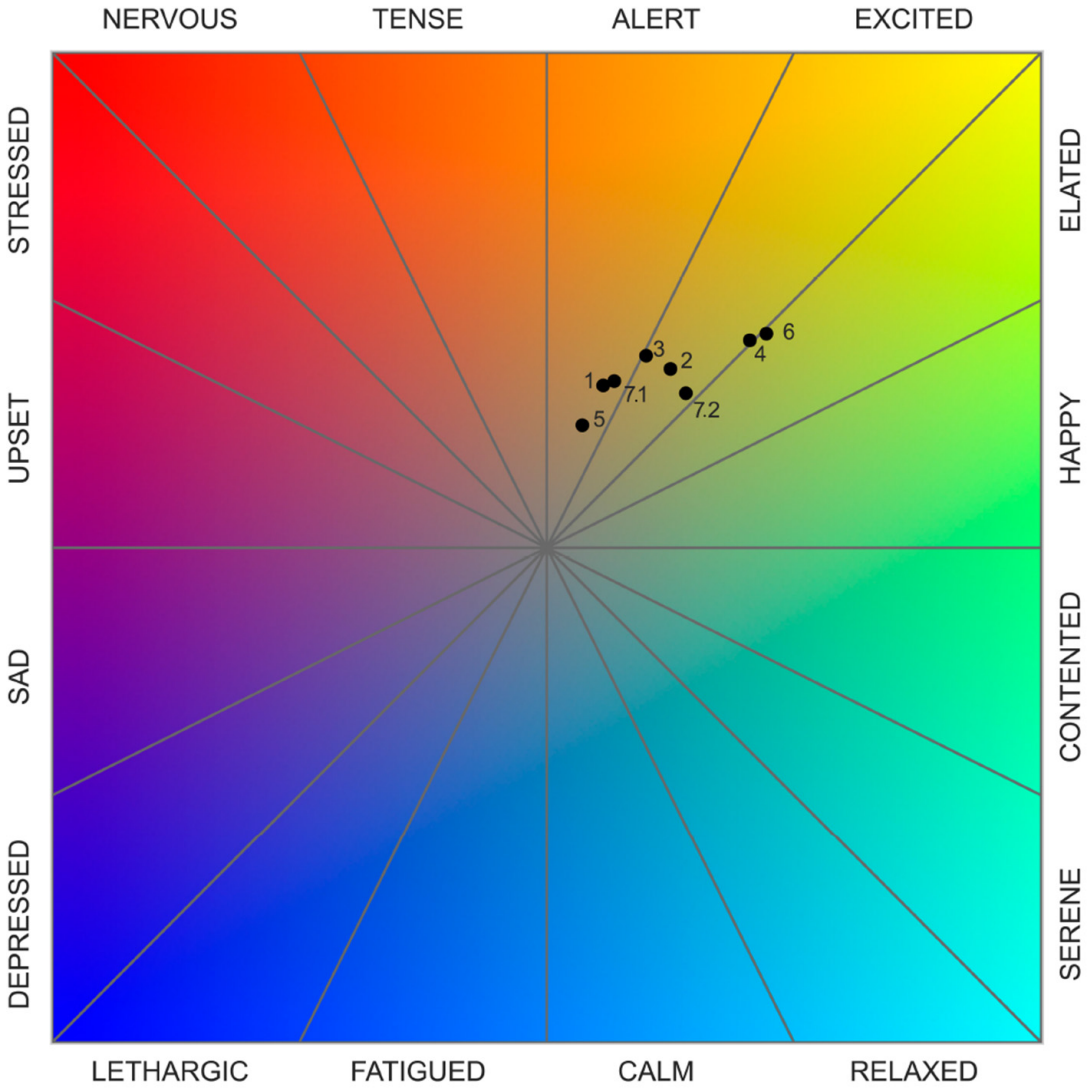
Emotions for each point according to the Russell circumplex model. Point distance from the center is proportional to the emotion intensity.

The spatial behavior of the participants, based on their Point of View and Targets, produced 8 clusters, confirming the instructions given by the facilitator. The mean values of valence and arousal of each cluster led to the computation of the position on the Cartesian plane of the circumplex model, in accordance with Russell and Pratt. The 8 evaluations are all included in the quadrant characterized by positive valence and high arousal, namely including emotions labeled as elation, excitement, and alertness; despite this similarity, they exhibit different degrees of intensity, placing more intense reaction closer to the perimeter of the plane and the others closer to the center. Following the path of the participants, PoV_1 “Café” is characterized by an alert reaction with low-medium intensity, PoV_2 “Train Station” and PoV_3 “Crosswalk” show a medium intensity of excitement emotion, PoV_4 “Square Entrance' is among the most intense reactions and lies in the area between excitement and elation, PoV_5 “Archway Passage” is the least intense reaction and is perceived as alerting, PoV_6 “Square Corner Outlook” is the most intense reaction and lies in the area between excitement and elation, PoV_7A “Tramway Stop South” shows an alert reaction with low-medium intensity, and PoV_7B “Tramway Stop East” has a low-medium intensity of excitement. Figure 2 includes the pictures corresponding to the urban environment explored in each PoV, Figure 8a emphasizes the intensity of the emotional reaction, Figure 8b depicts the geographic distribution of the data in the area corresponding to the actual average spatial behavior of participants' clusters, and Figure 9 shows the values on the cartesian plane.

For the arousal dimension, ANOVA remained significant (*p* < 0.05) even after Huynh-Feldt correction. Pairwise Bonferroni-corrected comparisons showed significant differences only between PoVs 4 and 5 (*p* < 0.01) and PoVs 5 and 6 (*p* < 0.05). The ANOVA result remained highly significant (*p* < 0.001) when considering the valence dimension as well. Pairwise comparisons (Bonferroni-corrected) revealed a more complex pattern of differences. PoV 1 differed significantly from PoVs 4, 6, and 7B; PoV 2 differed from PoVs 4, 6, and 7A; PoV 3 from PoVs 4 and 6; and PoV 4 from all other PoVs except PoVs 6 and 7B. In addition, PoV 5 differed significantly from PoVs 4, 6, and 7B; PoV 6 from PoVs 1, 2, 3, 5, and 7A; PoV 7A from PoVs 2, 4, 6, and 7B; and PoV 7B from PoVs 1, 5, and 7A.

In general, the results show a substantial difference in emotional experience between the different PoVs, with a stronger effect observed in the valence dimension compared to arousal. In particular, PoVs 4 (“Square Entrance”) and 6 (“Square Corner Outlook”) were perceived more positively, while PoVs 5 (“Archway Passage”) and 7A (“Tramway Stop South”) were associated with more negative emotional responses.

Table 3 shows the Pearson correlation coefficients computer between pairs of physiological and psychological variables. Significant correlation (either positive or negative) was found between Valence and the physiological metrics ARR, LF/HF, SD1, and SDSD, indicating agreement between the trends in the physiological and psychological indices. On the contrary, the change scores of physiological arousal weren't found to significantly correlate with any of the physiological metrics.

**Table 3:** Pearson correlation coefficients between change scores of physiological variables, and arousal and valence.

**Physiological variable**	**Valence**	**Arousal**
ARR	–0.9411^*^	–0.5071
PDA	0.5161	0.6761
HF	–0.6983	–0.169
LF/HF	0.9411^*^	0.3381
LF	0.0304	0.5071
SD1	–0.8804^*^	–0.169
SD1SD2	–0.5161	–0.6761
SD2	–0.6983	–0.3381
SDRR	–0.6983	–0.3381
SDSD	–0.8804^*^	–0.169
SCL	0.5768	0.5071

^*^Denotes statistical significance (*p* ≤ 0.05).

## 4 Discussion

Table 4 provides a comprehensive overview in the trends in the autonomic activity and psychological valence and arousal over the different Points of View.

**Table 4:** Trends in the autonomic activity and in the psychological valence and arousal over the different Points of View.

**Targets**	**Autonomic activity**		**Psychological appraisal**	
	**Sympathetic**	**Vagal**	**Arousal**	**Valence**
PoV_1—Cafe	↓↓	↑↑	↑	~
PoV_2—Square	↓	↑	↑↑	↑
PoV_3—Crosswalk	↑	↓	↑*↑↑*	↑
PoV_4—Square entrance	↑↑	↓↓	↑*↑↑*	↑↑
PoV_5—Archway passage	↑	↓	↑	~
PoV_6—Square corner outlook	↑↑	↓	↑*↑↑*	↑↑
PoV_7A—Tramway stop south	↑	↓	↑	~
PoV_7B—Tramway stop east			↑	↑

Analysis of the overall trends of the most significant physiological metrics (HF power, SD1, and SDSD for vagal activation, and LF/HF and PDA for sympathetic activity) allows us to draw conclusions on how autonomic activation varies throughout the guided walk. In particular, the initial two PoVs (PoV_1, “Cafe,” and PoV_2, “Train Station”), photographed in Figures 2a, b are characterized by predominantly vagal activation, indicating lower levels of arousal and neutral valence. These two points, and the walking sections connecting them, are situated in front of the Milano Greco-Pirelli train station, an open space that offers a rather unobstructed view of the sky, which is known from literature (Zhang et al., 2021) to promote relaxation. Arousal levels tend to increase when moving toward the Piazza della Scienza, as indicated by LF/HF and PDA trends. In particular, PoV_6 (“Square Corner Outlook”) appears to be the location that elicited the strongest sympathetic response in the experimental subjects. PoV_4 (“Square Entrance”) seems to be characterized by similar autonomic reactions, although the statistical significance of the considered metrics is weaker at this PoV. These two observation points (PoV_4 and PoV_6, photographed in Figures 2d, f) are located at opposite ends of the main square, and their directions of view pointed toward the center of Piazza della Scienza, offering a similar view, which explains the similar physiological reactions. Furthermore, the architectural elements there feature tall red buildings that enclose the square, limiting the portion of visible sky: all these elements are known from literature to have a negative effect on relaxation. PoV_6 (“Square Corner Outlook”) also features a rest area with benches that promoted social interactions, further increasing the arousing effects of this location. In contrast, PoV_5 (“Archway Passage,” photographed in Figure 2e) exhibits a notably different behavior than PoV_4 and PoV_6, despite being located between the two points. Interestingly, PoV_5 (“Archway Passage”) faces an archway passage through the university buildings that connects Piazza della Scienza to the other square around which the campus develops, Piazza Archway Passage. This passageway, at the time of this study, was partially blocked by a construction barrier related to inactive renovation works. Such a duller view, lacking the natural and social elements that are visible from the nearby PoV_4 and PoV_6 (trees and the social areas) can, at least partially, explain the observed decrease in sympathetic activity and valence that characterizes PoV_5.

The findings from the Emotional Index analyses suggest visible trends in a variety of emotional states as recorded during the guided walk. While these trends are striking, they don't always reach statistical significance. In particular, values of β_*EI*_ corresponding to the discrete Dominant Emotion “happiness” were measured with the highest relative frequency (approximately 30%) at PoV_3 (“Crosswalk”). A secondary peak centered around the “upset” discrete emotion is visible throughout the entire walk. The relative frequency with which this emotion is recorded increases gradually after leaving PoV_1 (“Cafe”), reaching its maximum value at PoV_4 (“Square Entrance”), before again in favor of emotional states characterized by higher valence and lower arousal. Traditional boxcharts are not well suited to represent the distribution of either the Emotional Index or the peak β_*EI*_ angle, due to their angular nature constrained within the [0, 2π] interval, where the endpoints 0 and 2π correspond to the same emotional state of “pleasantness'. Furthermore, the presence of multiple peaks and the non-normal, skewed distribution of both indexes provide additional justification for why traditional boxcharts are inadequate for representing these metrics.

The Russell-based polar histogram provides an alternative representation of the β_*EI*_ metric that allows us to appreciate different clusters of psychophysiological responses, without compressing the circular indexes into ill-fitted boxes. As visible in Figure 6, “happy” appears to be the dominant emotion during each PoVs. Secondary peaks are also present: the final PoV, “Tramway Stop,” in particular, displays an important secondary peak at “upset.” This specific PoV is the only one to feature two directions of view: it may be possible that the two peaks refer to the two specific directions of view, with the subjects mostly feeling happiness when looking in one of the two directions and upset when observing the other. However, such conclusion cannot be easily verified, as the sub-segments corresponding to each direction of view are not easily identified and separated within each recording. This physiological result is partially consistent with the physiological data: the two targets are analogous in terms of measured arousal, but not in valence. In particular, PoV_7B has a higher reported valence, which, in combination with the arousal, places the Target on the Russell plane at the boundary between “excited” and “elated.” PoV_7A, on the other hand, sits in the “alert” area.

With regard to the psychological assessment, both descriptive and variance analyses reveal consistent and interrelated patterns. They indicate that participants' emotional responses vary significantly depending on the point of view (PoV) presented, with the most marked contrasts found between PoVs 4 and 6 on one side, and PoVs 5 and 7 on the other. PoVs 4 and 6 are generally associated to more positive emotional dimensions, particularly *excitement* and *elatedness*, and higher levels of emotional intensity. Unsurprisingly, these locations correspond to spatial contexts that embody two key elements well-known to enhance perceived pleasantness and urban activation: opportunities for social interaction and the presence of green areas. The visual targets of both PoVs include the symbolic heart of the site—a partially secluded square surrounded by university buildings, enriched with vegetation and urban furnishings (mainly benches) that could support informal socialization among students or workers. These findings are fully consistent with the existing literature on environmental preference and pleasantness, which highlights that, particularly at the urban scale, small-scale green spaces (Nordh et al., 2011; Weber and Trojan, 2018) and furnished areas supporting nearby social interaction (Anderson et al., 2017) are generally preferred over other types of environments. However, the locations within the case study do not exhibit a sufficient degree of natural density to trigger effects such as attention restoration (Kaplan and Kaplan, 1989; Kaplan, 1995) or stress reduction (Ulrich et al., 1991). As a result, they fall within a moderately high range of perceived pleasantness and psychophysiological activation. Moreover, it can be hypothesized that partial seclusion and visual mastery contribute to the perception of pleasantness—at least during daytime hours, when the data collection took place—in line with the principles of Prospect and Refuge Theory (Stamps, 2008). In contrast, PoVs 5 and 7 evoke low to moderate levels of *alertness*, which can be explained by the distinctive features of each setting. PoV 5 offers a view toward the periphery of the square, obstructed by a visually disordered construction site. PoV 7 is instead located at the Tramway Stop stop, under a deteriorating concrete canopy that partially overhangs the waiting area. In particular, target 7A includes only buildings and urban infrastructures, with no natural or social element; furthermore, the concrete canopy completely obstructs the view of the sky. These aspects likely contribute to a heightened state of alertness among participants for Target 7A. In contrast, the same PoV 7 shows a slightly more positive experience for Target 7B, which includes trees and a broader view of the sky, shifting toward excitement due to increased valence. A comparable level of *alertness*, also within the low-to-moderate range, is associated with PoV 1. This location is set in the square in front of the main train station—a typically crowded space marked by continuous pedestrian and vehicular movement. In this case as well, the data appear to align with the literature on environmental preference. Locations with lower visual order (Kaplan, 1987; Ewing and Handy, 2009) or characterized by recurring environmental stressors (Cohen, 1991)—such as Tramway Stop noise, fast-moving individuals, or groups—tend to generate a heightened sense of alertness, consequently, reduced pleasantness. The absence of a strong emotional intensity, though seemingly counterintuitive, can be explained by the fact that these distressing elements (traffic, noise, and crowds) are typical features of the urban environment—and of this specific area in particular. Their presence is not perceived as sufficiently intense to evoke a high risk of personal space invasion or physical threat. In essence, these are habitual stressors, capable of producing a moderate state of alertness without triggering substantial psychophysical activation.

As mentioned in Results, Emotional Index paragraph (Section 3.1.2), psychological responses were grouped around the boundary between the “excited” and “elated” sectors of the Russell plane. Compared to the consistent “happy” psychophysiological dominant emotion, these results appear to be coherent in terms of the concentration of the response around a single emotion, but skewed toward higher levels of arousal. Partial discrepancies between physiological and psychological measures of emotions and mood have been reported in previous environmental evaluation studies, both in controlled laboratory settings (Chen et al., 2024; Hsieh et al., 2023) and during real-world walks in physical environments (Cao et al., 2023; Neale et al., 2022; Yin et al., 2023). For instance, (Chen et al. 2024) report that simulated transitions from built to natural environments and vice versa are accompanied by clear psychological changes, including dimensions of tension and vigor which can be conceptually equated to arousal (Russell and Barrett, 1999), whereas the physiological measures recorded during these transitions do not show significant changes. (Hsieh et al. 2023) further support this picture by showing that low-decibel waterscapes produce significant decrease in the psychological arousal dimension, whereas both low- and high-decibel waterscapes exert an influence on physiological indices. Despite a stronger effect of high-decibel waterscapes in moderating the autonomic nervous system, these results again indicate that the two domains may respond differently to the same environmental manipulation. A similar pattern emerges in studies that combine physiological and psychological indicators in different types of outdoor settings. (Cao et al. 2023) report that, across eight places and under both sunny and cloudy conditions, the psychological component of emotions measured through PANAS shows more marked pre-post changes than the corresponding physiological measures, suggesting a lack of full alignment between psychological and physiological components even when arousal is not directly emphasized by the psychological scale. (Neale et al. 2022) found that vigor increases when participants walk in an urban green environment and decreases in an urban gray condition, while physiological stress outcomes such as HRV also differ between conditions but with small effect sizes. Finally, (Yin et al. 2023) showed that, after 30-min walks, changes in heart rate, LFa/HFa ratio and mean arterial pressure did not support an association between blue space and physiological relaxation, whereas, on the psychological side, the presence of blue space was linked to greater improvements in mood states such as vigor. Furthermore, it is worth noting that these studies do not provide direct correlation analysis between psychological and physiological measures, hence the interpretation of the results remains largely descriptive. Taken together, these findings converge with our results in suggesting that emotional arousal, when assessed through self-report, can display systematic and interpretable patterns that are not always mirrored in physiological indices, reinforcing the view that psychological and physiological measures capture only partially overlapping facets of the emotional response to environmental settings.

A general explanation for the divergence observed in our study lies in the different levels of processing involved. While physiological responses often reflect pre-conscious or automatic affective reactions, self-reported emotions depend on conscious appraisal and introspection. This distinction becomes particularly relevant when assessing complex environments such as urban settings, where simultaneous exposure to multiple stimuli may elicit both positive and negative emotions, making physiological data less unidirectional. At the same time, such emotional variability may not be fully captured by psychometric tools, which offer only a snapshot of the individual's overall experience at a specific point in time. It is also important to highlight that in several previous studies, physiological and psychological data were collected before and after environmental exposure (e.g., Cao et al., 2023; Yin et al., 2023), thus misaligned with the moment of actual experience: this may have resulted in more consistent but less ecologically valid data. These considerations further support the strength of our approach. The use of multi-modal and temporally synchronized measurements, enabled by digital tools, allows for a more accurate and ecologically valid assessment of the complexity of emotional responses to urban spaces. In doing so, it overcomes the limitations caused by what (Ulrich et al., 1991) termed the “temporal non-congruence of the different measurement modes.” This offers a more nuanced picture of people's experience in urban contexts to inform decision makers. In addition, a histogram provides a clear and immediate visual representation of the emotions recorded, making it more accessible to those without a technical background. In the context of the platform where the plot is to be integrated, alongside cartographic maps displaying psychological reactions clustered by spatial distribution, this integration is crucial: urban planners must be able to quickly obtain a summary of the most pertinent information regarding the location of interest, considering both psychophysiological reactions and urban morphology along with environmental data. Our Polar Plot Representation of Emotional State seeks to condense the intricate phenomenon of physiological emotional reactions into an easily comprehensible visual format that meets these needs.

An important clarification should be made regarding the relevance and interpretation of the emotional index. It is essential to underscore that relying solely on a single index could lead to an incomplete interpretation of emotions, thus highlighting the necessity of integrating multiple physiological and psychological measures in our assessments. The evaluation of emotional valence through physiological measures, in particular, remains an unsolved challenge in psychophysiology and there is no absolute consensus among researchers on its most effective physiological correlates. Therefore, we chose to ascribe to Vecchiato's model (Vecchiato et al., 2014) for the computation of the Emotional Index, which has been previously validated in environmental studies (Bruno et al., 2024), while acknowledging its limitations.

## 5 Conclusion

Overall, our results highlight the importance of fine-grained spatial analysis in studies investigating people's reactions to urban settings, demonstrating that, even in pleasant environments, the affective landscape is far from uniform. The integration of spatial clustering and affective mapping, including physiological and psychological measures, presents a promising methodology for identifying emotionally salient areas both in need of intervention and acting as protective factors. From a planning and design perspective, these findings reinforce the necessity of context-sensitive approaches that consider the emotional impact of place-based interventions, which are part of a broader evidence-based approach to urban planning for promoting citizens' health (Bruno et al., 2024). The protocol we developed and tested in the context of this pilot study validated the possibility of measuring psychophysiological reactions to urban environments by recording physiological measures.

In particular, the Emotional Index, alongside with other more traditional indexes derived from Electrodermal Activity and Heart Rate, can offer precious insights into the way people perceive the environments that surround them. This new information can be used by urban planners to guide the development or renovation of urban areas in ways that maximize the physical and psychological wellbeing of citizens. The application of the experimental protocol to the campus of the Universitá di Milano Bicocca after the completion of the renovation works will offer the double opportunity to further validate our methodology and to evaluate potential changes in the psychophysiological reaction to the new appearance of the Scienza square.

## Data Availability

The raw data in aggregated form supporting the conclusions of this article will be made available by the authors, without undue reservation.
